# Not like night and day: the nocturnal letter-winged kite does not differ from diurnal congeners in orbit or endocast morphology

**DOI:** 10.1098/rsos.220135

**Published:** 2022-05-24

**Authors:** Aubrey Keirnan, Trevor H. Worthy, Jeroen B. Smaers, Karine Mardon, Andrew N. Iwaniuk, Vera Weisbecker

**Affiliations:** ^1^ College of Science and Engineering, Flinders University, Adelaide, SA, Australia; ^2^ Department of Anthropology, Stony Brook University, NY, USA; ^3^ Centre of Advanced Imaging, The University of Queensland, St. Lucia, QLD, Australia; ^4^ Department of Neuroscience, University of Lethbridge, Lethbridge, Alberta, Canada

**Keywords:** letter-winged kite, nocturnal, vision, skull, evolution, birds of prey

## Abstract

Nocturnal birds display diverse adaptations of the visual system to low-light conditions. The skulls of birds reflect many of these and are used increasingly to infer nocturnality in extinct species. However, it is unclear how reliable such assessments are, particularly in cases of recent evolutionary transitions to nocturnality. Here, we investigate a case of recently evolved nocturnality in the world's only nocturnal hawk, the letter-winged kite *Elanus scriptus*. We employed phylogenetically informed analyses of orbit, optic foramen and endocast measurements from three-dimensional reconstructions of micro-computed tomography scanned skulls of the letter-winged kite, two congeners, and 13 other accipitrid and falconid raptors. Contrary to earlier suggestions, the letter-winged kite was not unique in any of our metrics. However, all species of *Elanus* have significantly higher ratios of orbit versus optic foramen diameter, suggesting high visual sensitivity at the expense of acuity. In addition, visual system morphology varies greatly across accipitrid species, likely reflecting hunting styles. Overall, our results suggest that the transition to nocturnality can occur rapidly and without changes to key hard-tissue indicators of vision, but also that hard-tissue anatomy of the visual system may provide a means of inferring a range of raptor behaviours, well beyond nocturnality.

## 1. Introduction

The ecological diversity of birds is matched by a great variety of visual adaptations, ranging from species with excellent colour discrimination [1] and UV wavelength [2] detection to species that are almost blind [3]. The capacity of birds to adapt to new visual environments is also reflected in the evolution of behavioural shifts in activity patterns from diurnality to nocturnality [4–7]. Some species, such as the nocturnal kiwi (*Apteryx* spp.), have reduced their reliance on vision and depend more on tactile and olfactory information [3,8]. Other species, such as owls, nightjars and the Oilbird (*Steatornis caripensis*), rely on vision for foraging and other behaviours and see well in low light [9,10].

The evolution of nocturnality in birds is associated with a suite of anatomical changes [3,4,11–13]. The size of the eye corresponds with the number of photoreceptors in the retina [5], making large eyes advantageous in nocturnal species that rely on vision [4,14]. Within the retina, a higher proportion of photoreceptors to ganglion cells results in higher visual sensitivity, but lower acuity [5,15]. This higher retinal summation allows nocturnal species to see under low-light conditions, albeit with lower acuity. Although retinal summation is best determined from microscopic examination of the retina, it can be estimated in birds by comparing the orbit diameter (reflective of retinal area) with the diameter of the optic foramen (reflective of the number of retinal ganglion cells) [16]. Thus, nocturnal species tend to have small optic foramina relative to orbit diameters [16,17].

**Figure 1.**
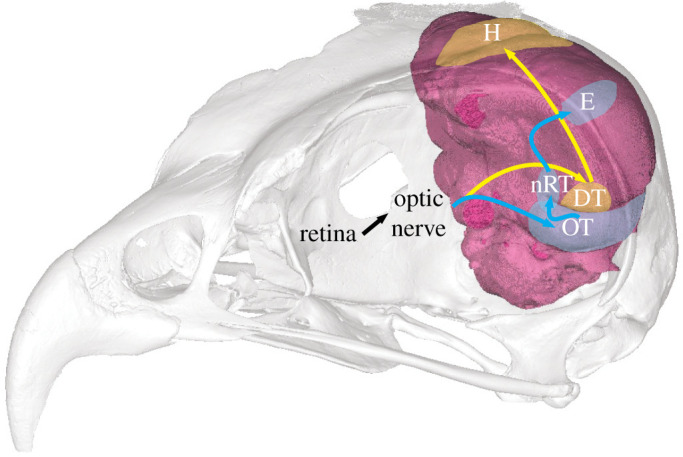
Letter-winged kite (*Elanus scriptus*, ANWC B30263) skull and endocast produced from µCT scanned skull in Materialise Mimics (v. 22.0) with approximate layout of two visual pathways. In blue is the tectofugal pathway (OT, optic tectum; nRT, nucleus rotundus; E, entopallium) while yellow is the thalamofugal pathway (DT, dorsal thalamus; H, hyperpallium). Adapted from Klose *et al*. [19] and Wylie *et al*. [20].

Visual information is processed along three different pathways in the avian brain: accessory optic, thalamofugal and tectofugal pathways [18]. There are differences in the sizes and morphology of the thalamofugal and tectofugal pathways in most nocturnal birds, relative to diurnal ones. The thalamofugal pathway, which projects from the retina to the dorsal thalamus and onto the hyperpallium ([18]; figure 1), is enlarged and has more prominent layers in owls and three other nocturnal species: Feline owlet-nightjar (*Aegotheles insignis*), tawny frogmouth (*Podargus strigoides*) and oilbird (*Steatornis caripensis*). This is thought to reflect a larger amount of visual processing associated with stereopsis in these species, rather than acuity [12]. The tectofugal pathway, which projects to the optic tectum, nucleus rotundus and up to the entopallium [18,20], comprises the majority of retinal afferents in birds [21] and exhibits a different pattern to the thalamofugal pathway in nocturnal birds. Rather than being enlarged, the tectofugal pathway tends to be smaller in most nocturnal species ([3,11,22], which we hypothesize is likely due to higher retinal summation.

The anatomical changes in the eyes and brain outlined above apply to most of the nocturnal species examined to date, but nocturnality has evolved independently multiple times in avian evolution [4,16]. Determining whether anatomical changes associated with nocturnality are similar across lineages, habitats and foraging behaviours can reveal how these changes arise, what selection pressures cause them, and yield insights into the sensory abilities of less-well studied species. Of course, obtaining eyes and brains of rare species is not always feasible and detailed anatomical analysis is labour-intensive, but some aspects of their anatomy can be estimated from skulls. Orbit dimensions and optic foramen size closely approximate that of the eye and the optic nerve [4,16]. Key components of both the tectofugal and thalamofugal pathways are also visible on the surface of the avian brain, and their surface areas are correlated with the underlying brain region volumes [23,24]. The wulst is the dorsal extension of the hyperpallium of the thalamofugal pathway and is visible on the dorsal surface of the brain. As mentioned above, it is greatly enlarged in owls and other nocturnal birds, and it is associated with binocular vision and depth perception [12,13]. The optic tectum is housed in the optic lobes that protrude on the ventro-lateral aspect of the avian brain [20,25].

Examining orbit and endocast morphology of birds can clearly provide insights into the evolution of the visual system in nocturnal species for which histological data cannot be obtained. For example, nocturnality evolved at least twice within parrots: in the New Zealand kakapo (*Strigops habroptila*) and in the Australian night parrot (*Pezoporus occidentalis*). The kakapo underwent a reduction in the size of the optic foramen and the tectofugal pathway relative to other parrots, but lacks enlargement of the thalamofugal pathway, suggesting it has relatively higher visual sensitivity and lower acuity [11]. Brains have yet to be examined of the night parrot, but it too shows evidence of higher sensitivity and lower acuity compared to similarly sized diurnal parrots [17]. This has implications for conservation efforts, as fences and powerlines may pose greater threats to the night parrot than their diurnal relatives [17]. Conversely, if a nocturnal species is highly sensitive to low light, artificial light and light pollution could negatively impact their orientation, reproduction and predation susceptibility [26].

Among the birds of prey (owls, falcons, hawks and vultures), owls have been the focus of numerous anatomical studies [4,27,28], but the other groups within this paraphyletic assemblage of predatory birds have received less attention. In particular, hawks (Accipitriformes) and falcons (Falconiformes) have exceptional vision [29] and are typically diurnal due to their foraging style of capturing small, fast prey from great heights [30,31]. Several raptor species can hunt at dusk, and anecdotal reports suggest that some can also hunt at night. However, the only species that appears to be primarily active at dusk and at night is the letter-winged kite (*Elanus scriptus*). This small hawk is endemic to Australia [32] and is believed to occupy a similar ecological niche to the barn owl (*Tyto alba*) [33]. Their populations seem to depend on rodent irruptions, which occur after heavy rain in arid regions of central Australia [32,33]. *Elanus scriptus* typically avoids towns and settled areas, living mostly in arid locations [32]. This habitat preference, along with their nocturnal activity pattern and nomadic movements, makes *E. scriptus* difficult to observe in the wild [34,35]. However, anatomical adaptations of *E. scriptus* to low-light conditions have been the subject of much speculation. Pettigrew (1982) suggested that the soft tissue anatomy of the eyes of *E. scriptus* resemble those of nocturnal birds more than of diurnal species, including its Australian congener, the black-shouldered kite (*Elanus axillaris*). Observations by Pettigrew [13], however, indicate that this species is still reliant on moonlight. The eyes of *E. scriptus* are also thought to be larger and more frontally orientated than those of its congener, *E. axillaris* [36], but actual measurements are lacking. The divergence between *E. scriptus* and the diurnal white-tailed kite *E. leucurus* is very recent, estimated at around the mid-Pliocene (3.3-3 MYA; [37]). The other species within the genus *Elanus* are diurnal, but have been suggested to exhibit ‘owl-like’ anatomical and behavioural traits [38]. Even *E. axillaris*, the Australian congener of *E. scriptus*, is opportunistically crepuscular, suggesting that some adaptations to low light are common to all members of the genus [39,40].

In this study, we use micro-computed tomography (μCT) of *E. scriptus*, two diurnal species of *Elanus*, and a set of hawk and falcon species with diverse hunting styles to ask whether the nocturnal lifestyle of *E. scriptus* is reflected in its hard-tissue anatomy. We specifically test whether *E. scriptus* has larger, more frontally oriented orbits [41,42], and if the relative sizes of the orbits, optic foramina, optic lobes and wulst differ from those of other species. If *E. scriptus* has evolved more sensitive vision than other hawks, we predict that it will have smaller optic foramina relative to orbit diameter and proportionately smaller optic lobes, as observed in other nocturnal species. Alternatively, if all *Elanus* kites share an ability to hunt under low-light conditions, a similar anatomical morphology differing from that of other accipitrids could be representative of the genus and not restricted to *E. scriptus*.

## 2. Methods

### 2.1. Species collection and scanning

We used μCT X-ray scanning techniques (henceforth μCT-scanning) to obtain high-resolution (20.5–34.9 um) images from 30 raptor skulls, all of which were sourced from museums within Australia. We included 16 raptor species, 12 of which belong to the Accipitriformes (hawks, eagles and kites) and the remaining three to the Falconiformes (falcons). Each species was represented by at least two individuals, apart from the little eagle (*Hieraaetus morphnoides*), the brahminy kite (*Milvus indus*) and the black-winged kite (*Elanus caeruleus*). We examined three specimens each of Letter-winged Kite (*Elanus scriptus*) and black-shouldered kite (*Elanus axillaris*). Scans were produced at multiple scanners and facilities: a Siemens Inveon PET-CT scanner at the Centre for Advanced Imaging; a Nikon XT H 225ST at the Tonsley Medical Imaging Centre and a SkyScan 1276 at Adelaide Microscopy. Access to one specimen of *E. caeruleus* (1850.8.15.159 from the Natural History Museum in London), first published in [43] and deposited in MorphoSource (Media ID 000092040), was provided by Prof. Roger Benson. A complete list of specimen numbers, data and scan resolution is provided in electronic supplementary material, table S1.

### 2.2. Endocast acquisition

**Figure 2.**
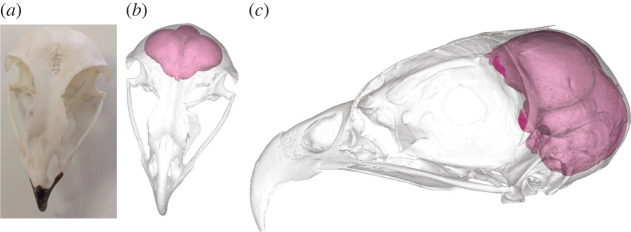
Different views of letter-winged kite (*Elanus scriptus*) skull (ANWC B30263) from photography and three-dimensional imaging software, Materialise Mimics (v. 22.0), with endocast visible. (*a*) Photograph of the dorsal view of the skull; (*b*) dorsal view of the reconstructed three-dimensional image of the skull in a similar position to the photograph showing the endocast; (*c*) lateral view of the reconstructed three-dimensional image of the skull and the endocast.

Endocast acquisition and all measurements except ‘neurocranial length’ (see below) were conducted by A.K. Scan images were reconstructed virtually using the three-dimensional imaging software Materialise Mimics (v. 22.0). Endocasts were segmented by digitally blocking all gaps (such as nerve foramina) in the skull and ‘flood-filling’ the braincase, following best practices as described in Balanoff *et al*. [44] and implemented in previous studies [17,45,46]. An example of an endocast of *E. scriptus* within the skull can be seen in figure 2. All CT scans and derivative surface files are publicly available on MorphoSource project ID 00000C918.

Some endocasts displayed slight damage to one side of the telencephalon, likely due to the cause of death. For these specimens, the undamaged side was digitally mirrored and registered with the surrounds of the damaged site using the ‘global registration’ functionality of Mimics. Examples are provided in the electronic supplementary material, figures S1 and S2. The endocast surface was measured with and without the blood vessels and the trigeminal ganglia that surround the optic lobes (if present; see electronic supplementary material, figure S3), with a linear regression revealing minimal differences for endocast surface estimates (Slope = 1; *R*^2^ = 0.999; *p* = 0.000). Either measurement can clearly be used, but we took the more cautious approach of using surface areas with vessels and ganglia removed.

To measure surface areas and volumes, the endocasts were imported into Materialise 3-Matic (v. 14.0) where the ‘brush’ tool was used to collect the surface area of the optic lobes (electronic supplementary material, figure S4) as defined by Early *et al*. ([23]; electronic supplementary material, figure S5) as an approximation of the volume of the optic tectum [23]. We also used the ‘brush’ tool to obtain approximate surface areas for the wulst region on the dorsal surface of the brains. Not all species had a clear distinction between the wulst and the remainder of the surface, making this measurement less reliable than the clearly defined optic lobes. However, we deemed the delineation of the wulst sufficient to determine if *Elanus* species were substantially different to compared species and therefore included the measurement.

### 2.3. Skeletal measurements

**Figure 3.**
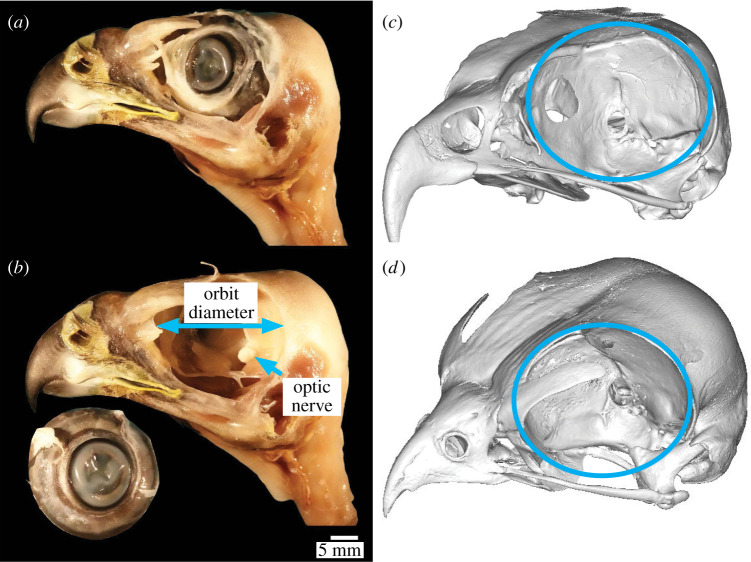
Orbit diameter definition and measurement. Images (*a*) and (*b*) provided by A.N.I. (*a*) Dissected Cooper's hawk (*Accipiter cooperii*) with the eye in position within the orbit and (*b*) with labels showing approximate positions for measurements. (*c*,*d*): three-dimensional skull reconstructions ((*c*), letter-winged kite NMV B30263, (*d*), nankeen kestrel QM O31414) with orbit diameter highlighted in blue.

To determine if eye size in *E. scriptus* differs from that of diurnal species, the diameter of each orbit was estimated using the ellipse tool of Mimics. The bony margins of the orbits that were used to take this measurement were verified as representing the eye diameter through dissections of a Cooper's hawk and a nankeen kestrel (figure 3). The eye spans the breadth and height of the bony orbit. The maximum distance between the anterior and posterior margins approximates the transverse diameter of the eye. The ellipse was also constrained by the dorsalmost extent of the orbit rim (figure 3*c*,*d*). To further ensure the accuracy of these measurements for the museum specimens examined, we compared our orbit diameters with the transverse diameters measured from the same species in Ritland [47].

Optic foramen diameters were measured in Mimics v. 22 (see examples in electronic supplementary material, figure S6) as a proxy for optic nerve size [16], which in combination with orbit diameter measurements can reflect the degree of retinal summation [5]. In the case of one specimen each (out of two) of *Haliastur sphenurus* and *Aviceda subcristata*, the left foramina were too damaged to measure. For these, we only provide measurements from the right side.

**Figure 4.**
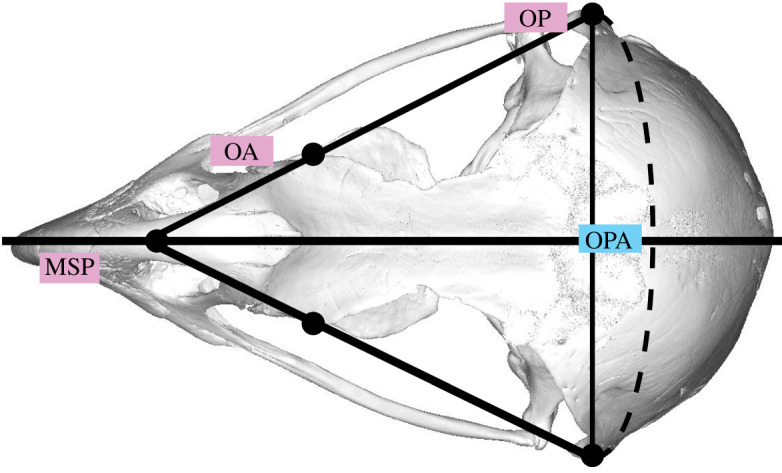
Dorsal view of a letter-winged kite (*Elanus scriptus*, NMV B30263) skull showing orbit convergence measurements and landmarks used to acquire them. OP, orbit posterior (and skull width); OA, orbit anterior; MSP, mid-sagittal plane; OPA, orbit posterior angle.

Last, we estimated orbital convergence. This is because *E. scriptus* has often been described as having ‘owl-like’ orbits that are more frontally oriented [34]. We combined methods from Duhamel *et al*. [48] and Borges *et al*. [49] and used Materialise Mimics angle, distance and plane tools. Orbital margin convergence (OMC) was calculated using the orbit posterior angle (OPA). As shown in figure 4, this angle is defined as that between the two points at the posterior most part of the orbital margin (orbit posterior; OP), intercepting the anterior most part of the front of the orbital margin (orbit anterior; OA) and meeting at the mid-sagittal plane (MSP) (figure 4).

### 2.4. Statistical analysis

The dataset of all measurements taken above, and the script to execute all analyses, are available on Github (https://github.com/VWeisbecker/LWK_Visual) and all analyses were done in R v. 4.1.2 [50]. We used the geometric mean of all linear distance measurements for each individual as a measure of isometric size across species [51]. However, as the geometric mean is designed to minimize the effects of shape and conceivably would generate a similar value for a short wide and a long narrow neurocranium, and so might exclude differences in relative size in more lateral zones, we also explored the use as a size proxy of a simple metric, neurocranial length, defined as the distance between the occipital condyle and the craniofacial hinge. Right and left side measurements were averaged in all cases, and data for species represented by several individuals were then averaged in the comparative analyses, but we show data for all specimens in graphs.

**Figure 5.**
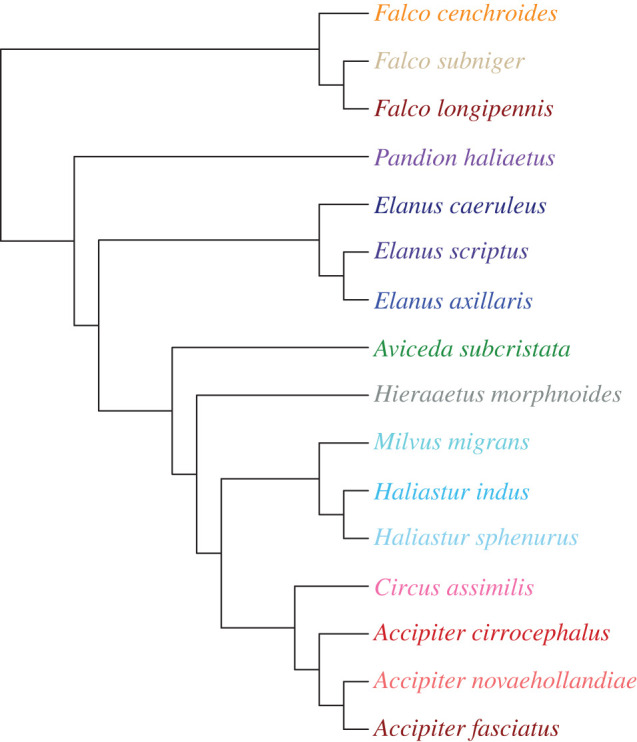
Phylogeny used here of 16 Australian raptor species. The colour scheme is used throughout this manuscript.

Statistical analyses were conducted within a phylogenetic framework. We initially reconstructed a 95% majority consensus tree from 1000 phylogenetic trees derived from http://birdtree.org [52], using the *ape* software package v. 5.5 [53]. This resulted in a polytomy for all three species of *Accipiter* and *Circus assimilis*, and a polytomy for the three species of *Elanus*. Using a 50% majority consensus tree resulted in the same topology with a better-resolved *Accipiter* and only a single a polytomy including *C. assimilis* and the species of *Accipiter.* We resolved this by placing *Circus* outside this genus, following the topology in Mindell *et al*. [37]. We resolved the genus *Elanus* by placing *E. axillaris* as the sister species to *E. scriptus* to the exclusion of *E. caeruleus*, consistent with Mindell *et al*. [37] and Starikov & Wink [54]. The tree was ultrametricized using Grafen's method [55] and is provided in figure 5. All analyses assumed Brownian motion evolution. Matching of tree and dataset was supported by the functionalities of the *geiger* software package v. 2.0.7 [56].

We assessed evidence of higher retinal summation in *Elanus* by asking whether the genus differs from the other species by having greater orbit diameters, smaller optic nerve diameters and smaller optic lobe surface areas relative to the geometric mean, whether the orbit diameters were larger relative to optic nerve diameters, and whether the optic lobes were smaller relative to the endocast surface area. We tested this using phylogenetic analysis of covariance (pANCOVA) function from the *evomap* software package [57] and phylogenetic generalized least-squares (PGLS) analysis as implemented in the *nlme* software package [58]. This allowed us to ask whether a model that includes a different intercept for the species of *Elanus* is significantly better than one that assumes the same intercept for all raptor species. Note that the pANCOVA implementation we use [59] employs standard least-squares procedures only (not maximum-likelihood optimization) and is therefore suitable for asking if a small number of species, or even one, differs significantly from the remainder of the sample. Last, we investigated if the species of *Elanus* are more like owls compared to other raptors in having greater optic convergence (more forward-facing eyes) compared to the other species, using phylogenetic ANOVA (pANOVA) as implemented in the *phytools* software package v. 07–9 [60]. We assessed the linear models that form the basis of our PGLS and pANCOVA models for normal distribution of residuals using the check_normality function of the *performance* software package [61].

We also visualized the relationships tested above by plotting the raw data for all individuals with phylogenetically informed 95% confidence intervals (which indicate the degree of uncertainty in the regression parameters) on each scatterplot. Where a pANCOVA was significant (or nearly so), we also plotted the prediction intervals (which predicts in what range a future observation will fall) as a visual assessment if species differed substantially from expected proportions. Note that, following their different use and interpretation, confidence intervals were calculated using the entire sample, and prediction intervals were calculated while excluding *Elanus* from the sample. Both confidence and prediction intervals were calculated following the procedures described by Smaers & Rohlf [59] using the *evomap* software package [57]. This was done because, since the procedures we use are based on standard least-squares procedures only, the low sample sizes make the pANCOVA results conservative. As such, the confidence and prediction intervals allow a visual assessment of whether a potential significant difference could be expected with larger sample sizes. Last, to determine if *E. scriptus* had more frontally orientated orbits than diurnal raptors, we created univariate plots of the orbit convergence measurements.

## 3. Results

**Figure 6.**
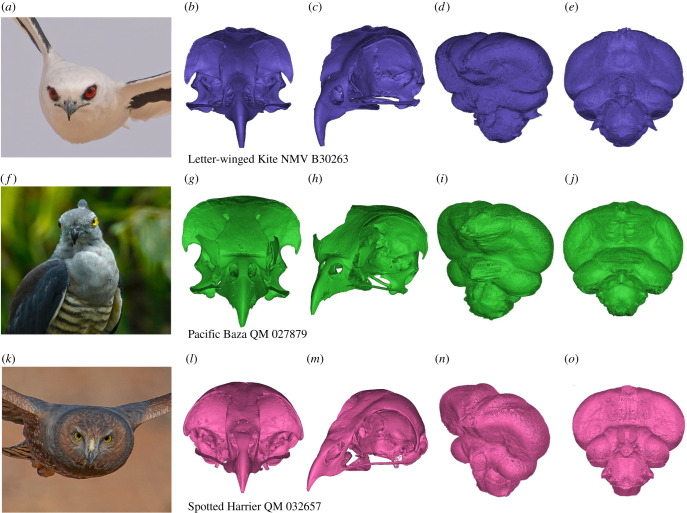
Distinct accipitriforms illustrated by their skulls (left) and endocasts (right). (*a–e*) Letter-winged kite (*Elanus scriptus*), (*f–j*) Pacific baza (*Aviceda subcristata*) and (*k–o*) spotted harrier (*Circus assimilis*). *Elanus scriptus* and *C. assimilis* images provided by Michael Jury of mykelphotography.com.au, baza image provided by Prof. Michael S. Y. Lee, skull and endocasts constructed in Materialise Mimics.

For a visualization of skull and endocast morphology of several species of particular interest, see figure 6. Our orbit measurements were comparable to earlier measurements on the same species by Ritland ([47]; electronic supplementary material, table S2). There was some intraspecific variation in some species, likely due to sexual size dimorphism [39]; this was greatest among individuals for the collared sparrowhawk (*Accipiter cirrocephalus*) (electronic supplementary material, figure S7). The museum record did not provide the sex for one specimen; however, this individual (QM 33127) was far larger than the other (QM 28021), which was recorded as a male. Female collared sparrowhawks can be nearly twice the size of males (125 g and 240 g, respectively; [32]). As the measurements of our unsexed specimen were 126–159% larger than the smaller male specimen and well within the range of sexual dimorphism for this species, we attributed it to a female. Endocast data have not often been presented for multiple individuals of a species, so to display intraspecific variability, we plotted all individual values on the graphs. However, the regression lines and confidence/prediction intervals on these graphs were based on PGLS analyses of datasets where intraspecific variation was averaged.

### 3.1. Residual normality of phylogenetic generalized least-squares models and use of neurocranial measurements

**Figure 7.**
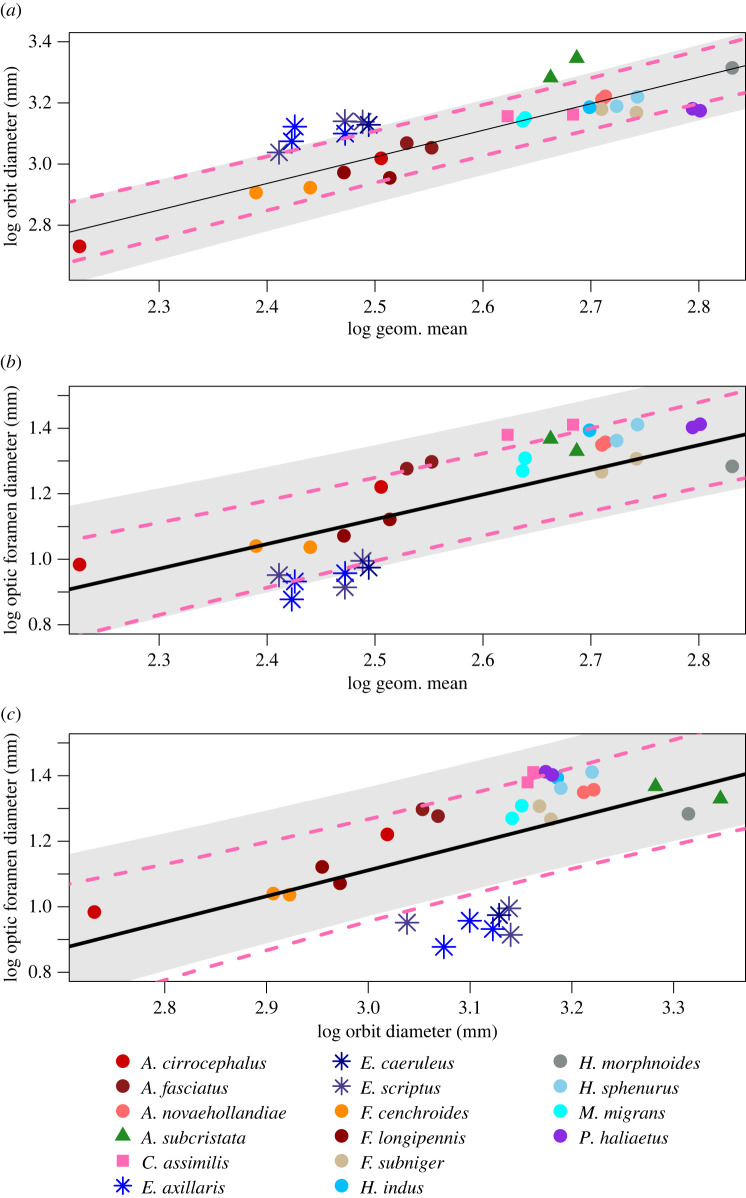
Bivariate plots of orbit diameter (*a*) and optic foramen diameter (*b*) against the geometric mean, and optic foramen diameter against orbit diameter (*c*). Points represent averages for both sides for all individual specimens. Regression lines and intervals drawn with coefficients from PGLS of species averages. Grey areas are the 95% prediction intervals (predicted values relative to size without *Elanus*), pink dashed lines delimit 95% confidence intervals for whole regression.

**Figure 8.**
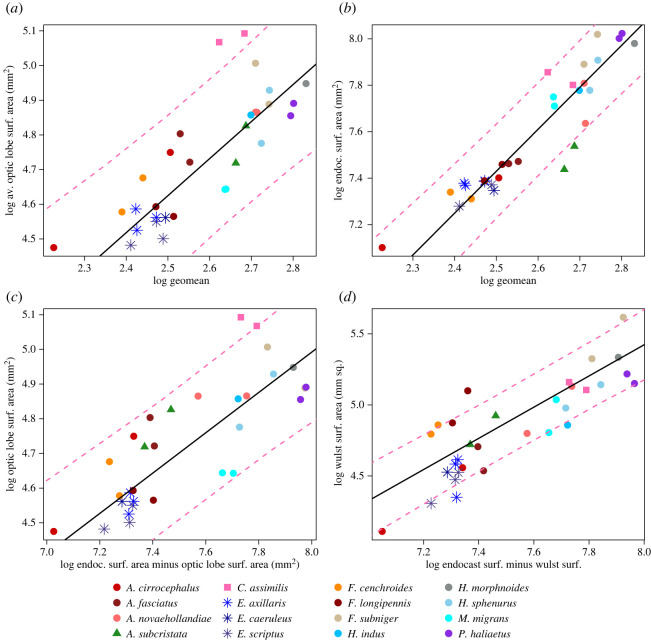
Bivariate plots of surface areas against the geometric mean for optic lobes (*a*) and endocasts (*b*), and surface areas against endocast surface area minus the respective optic lobe portion (*c*) and minus the wulst portion (*d*). Points represent averages for both sides for all individual specimens. Regression lines and intervals drawn with coefficients from PGLS of species averages. Pink dashed lines delimit 95% confidence intervals for whole regression.

Most models tested had normal distributions of residuals. However, three PGLS models and models with grouping factors revealed significant, but slight, deviations from normality. These were most likely driven by a small sample size overall coupled with several outlying values (tables 1 and 2; compare to scatterplot figures 7*a*,g, and 8*d*), which we note below as caveats. Analyses that used neurocranial length instead of the geometric mean yielded near-identical results, with the exception that *Circus* did not show a significant difference in optic lobe surface area relative to neurocranial length (electronic supplementary material, Results S1; this difference also did not explain much of the variation in the analyses using the geometric mean, see results below).

**Table 1.** Results from the PGLS regressions for multiple variables measured in the skulls of our sample. Slope, regression slope coefficient, *t* statistic; *p*, probability of no significant association between variables; Res. Norm, probability of normality of model residuals (a value below 0.05 indicates that the distribution of model residuals differs significantly from normal). Statistically significant (*p* < 0.05) results have been italicised. All variables were logarithm transformed.

dependent	predictor	slope	*t*	*p*	res. norm.
orb. diam.	geom. mean	0.87	13.791	0.000	0.262
opt. for.	geom. mean	0.755	7.948	0.000	0.224
opt. for.	orb. diam.	0.792	6.17	0.000	*0.014*
opt. L. surf	geom. mean	1.068	6.167	0.000	0.111
endoc. surf.	geom. mean	1.804	11.915	0.000	0.126
opt. L. surf.	endoc. surf.	0.581	7.798	0.000	0.954
wulst surf.	endoc.-wulst surf.	1.097	11.81	0.000	*0.039*

**Table 2.** Results from the pANCOVA analysis of log-transformed measurements. ‘Full model’ is the model assuming the same intercept for all species; ‘+*Elanus*/*Circus* group’, the model adding the suggestion that *Elanus* or *Circus* are distributed along a different intercept. d.f., degrees of freedom; sum sq, sum of squares; mean sum Sq, mean sum of squares, *F*, *F*-statistic; Pr (>*F*), probability that the model with different intercepts is better; Res. Norm, probability of normality of model residuals. Statistically significant (*p* < 0.05) results have been italicised.

dependent	predictor		d.f.	sum sq	mean sum Sq	*F*	Pr (>*F*)	res. norm.
orb. diam.	geom. mean	full model	3	0.0552	0.0042	4.5377	0.0528	*0.003*
		+ *Elanus* group	2	0.0745	0.0053			
opt. F. diam.	geom. mean	full model	3	0.1257	0.0097	4.4968	0.0538	0.311
		+ *Elanus* group	2	0.1691	0.0121			
orb. diam.	opt. F. diam.	full model	3	0.1963	0.0151	6.3392	*0.0257*	0.07
		+ *Elanus* group	2	0.292	0.0209			
opt. L. surf.	endoc. surf	full model	3	0.3626	0.0279	0.0888	0.7704	0.163
		+ *Elanus*	2	0.3651	0.0261			
opt. L. surf.	endoc. surf.	full model	3	0.2791	0.0215	4.0051	0.0667	0.33
		+ *Circus*	2	0.5746	0.041			
opt. L. surf.	geom. mean	full model	3	0.3621	0.0279	7.1729	*0.019*	0.729
		+ *Circus*	2	0.5618	0.0401			

### 3.2. Orbit and optic foramen diameters

Orbit and optic foramen diameters scale with the geometric mean (table 1 and figure 7), but note the PGLS model of orbit diameter versus optic foramen diameter did not have normally distributed residuals, likely due to the datapoints for *Elanus* (see below and figure 7*c*). There was no visible difference in the relationship between the orbit and optic foramen diameters between the nocturnal *E. scriptus* and its diurnal congeners; all elanine kites were grouped together in our plots (figure 7*a,b*). Elanine kites as a whole appear to have relatively large orbit diameters, but relatively small optic foramina compared to other species, although the pANCOVAs did not quite reach statistical significance (0.0528 and 0.0538, respectively, table 2 and figure 7*a,b*). However, these two trends do result in elanine kites having significantly smaller optic foramina relative to their orbit diameters than the other raptor species examined (figure 7*c* and table 2).

### 3.3. Endocast surfaces

The endocast and optic lobe surface areas scale with the geometric mean and each other (table 1 and figure 8). Note that the residuals of the wulst-endocast surface areas do not satisfy the assumption of normality. All of the elanine kites, including the *E. scriptus*, have optic lobe and wulst surface areas typical for their endocast surface area (figure 8*c,d*). The only species that was clearly outside the phylogenetic confidence intervals in terms of optic lobe area was the spotted harrier (*Circus assimilis*). This species' optic lobe surface area was much larger than expected for its endocast surface area and the geometric mean (figure 8*a,c*, pink points outside the phylogenetic confidence interval). This observation was partially supported by a follow-up pANCOVA; *C. assimilis* also has significantly larger optic lobes relative to the geometric mean, but not relative to endocast surface area (table 2).

### 3.4. Orbital margin convergence

**Figure 9.**
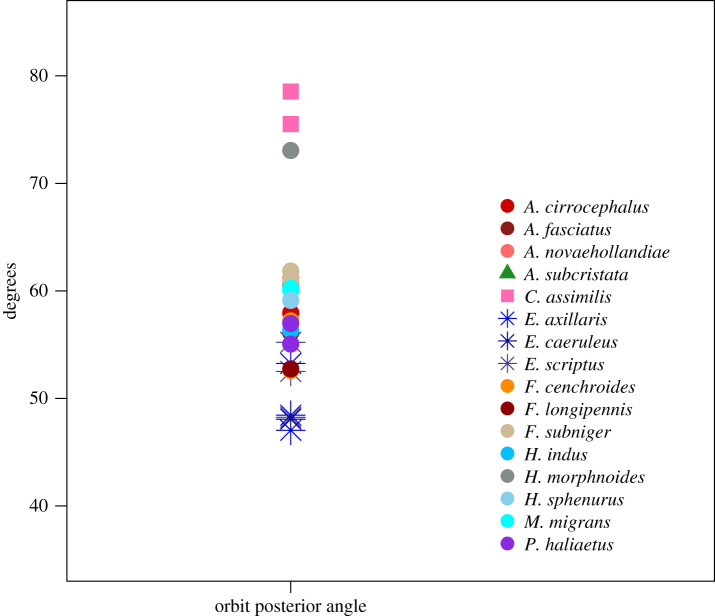
Comparison of orbit posterior angle which is used to measure the orbital margin convergence (measured as in Menegaz & Kirk [14] and Duhamel *et al*. [48]).

The convergence of orbital margins of *Elanus* species neither differed from the remainder of the sample, nor within the genus; the average convergence of *E. scriptus* orbits was only 2.41° larger than for *E. axillaris*, but neither species are distinct from other diurnal species (figure 9). On the other hand, the spotted harrier (*Circus assimilis*) and Pacific baza (*Aviceda subcristata*) represented extremely wide and narrow orbital convergence angles, respectively, which a follow-up pANOVA confirmed as a significant difference (table 3; note that the baza difference is very close to the significance cut-off of 0.05, however).

**Table 3.** Results of a PhylANOVA testing whether species in the genus *Elanus*, *Circus assimilis* or *Aviceda subcristata* have significantly different orbit convergence angles compared to the other species. Statistically significant (*p* < 0.05) results have been italicised.

Genus	MeanSq	*F*	*p*
*Elanus*	210.1902	2.930738	0.392
*Circus assimilis*	409.8186	7.132239	*0.005*
*Aviceda subcristata*	366.2063	6.045485	*0.033*

## 4. Discussion

We asked if key skeletal anatomical indicators of nocturnality were present in the visual system of the only nocturnal hawk, the letter-winged kite (*E. scriptus*), compared to several other hawks, including two congeners, and falcons. Rather than a specific difference in *E. scriptus*, we found that all three sampled species of *Elanus* have small optic foramina relative to orbit diameter compared with other species. By contrast, neither *E. scriptus* nor other species of *Elanus* differed in the relative size of the optic lobes or the wulst. Unexpectedly, the spotted harrier (*Circus assimilis*) emerged as having enlarged optic lobes and more convergently oriented orbits. Overall then, we found no specific anatomical changes associated with nocturnality in *E. scriptus*, but the diversity in our measurements supports recent suggestions that hawks and falcons are not a uniform group and possess high variation in sensory abilities [62].

The lack of difference among *Elanus* species adds to existing evidence that that the switch from diurnal to nocturnal activity may not involve extensive or gross changes in skeletal visual system anatomy. For example, the night parrot [17] has smaller optic foramina and optic lobes compared to diurnal parrots, but no differences in relative orbit size. Similarly, the nocturnal swallow-tailed gull (*Creagrus furcatus*) does not differ in orbit size, eye size/shape or orbital convergence compared to other gulls [63], despite foraging more during darker periods of the lunar cycle [64]. Thus, evolutionary shifts in activity pattern are not always reflected in anatomical differences in the skull. Of course, not all anatomical changes in the nervous system are reflected in osteology. In the case of *E. scriptus*, the F-ratio (eye length/corneal diameter) and the pecten folds appear closer to those of other nocturnal birds (e.g. owls, frogmouth, owlet-nightjar and oilbird) than to the black-shouldered kite (*E. axillaris*) and other diurnal birds [34]. The larger aperture relative to eye length would allow more light to be collected by the retina of *E. scriptus*, which is clearly advantageous for nocturnal foraging. Higher rod : cone ratios within the retina enable low-light vision [3,34] and would not result in changes in the orbits or other parts of the skull. Thus, *E. scriptus* might have anatomical adaptations for nocturnal foraging in the eye/retina that are not found in other hawks, including other *Elanus* kites. This hypothesis will require research on freshly fixed specimens of *E. scriptus*, *E. axillaris* and other accipitrid species.

Not only were all three *Elanus* kites similar to one another across our measurements, but they also all had comparatively large orbits and small optic foramina (figure 7), and significantly smaller foramina relative to orbit diameter. However, the orbits were not more convergently oriented (larger convergent angles), contrary to descriptions of *Elanus* kites possessing an ‘owl-like’ appearance [38,65]. The optic foramen is a proxy for the optic nerve, so a smaller foramen reflects fewer retinal ganglion cell axons and a higher degree of retinal summation (ratio of photoreceptors to retinal ganglion cells). Higher retinal summation enables more sensitive vision [66]. We therefore propose that all *Elanus* kites share higher retinal summation than the other raptor species examined, facilitating the shift of *E. scriptus* into a nocturnal activity pattern with fewer anatomical changes.

Why all *Elanus* kites would share this anatomical trait is uncertain, but it does fit with the observation that species of *Elanus* share some similarities with owls [38]. For example, unlike other hawks, *Elanus* kites have owl-like barbules on the upper surface of their primary and secondary feathers, which would enable silent flight [67]. It would be interesting to assess whether *Elanus* kites also share some of the unique sclerotic ring anatomy of owls, which result in a tubular eye that enhances night vision [68]. Sadly, sclerotic ossicles are not available in sufficient numbers for comparative analysis because they are mostly missing in osteological specimens. Ecologically, *Elanus* kites also share with several owl genera a dietary reliance on small rodents in grasslands and other open habitats [69]. Intriguingly, this food and habitat preference is also shared with harriers; in fact, both *Elanus* and harrier species use a unique hunting style of quartering [65] (low, continuous forward flight over tall grasses and marshes with the beak pointed downwards [70]). However, species of *Elanus* hover more than they quarter or perch-hunt [69]. It has even been suggested that species of *Elanus* might use auditory cues to localize prey when hovering [38], but their hearing abilities have yet to be tested. Although many other hawk species will occasionally hunt at dawn and dusk [71], crepuscular and moonlit night hunting behaviour is commonly reported in *Elanus* kites [65,72,73], which might have driven a shift in retinal summation such that all species within the genus have higher visual sensitivity than other hawks.

Unexpectedly, the Pacific baza (*Aviceda subcristata*) was unusual in having the largest orbits, both relatively and absolutely, and striking laterally positioned orbits, which gives them a pigeon-like appearance (figure 6*f*); otherwise, while it had a small endocast surface area for its overall size, it was not conspicuously different to other raptors in any of the other visual system traits we assessed. The baza primarily hunts insects and frogs in trees, often foraging using a still-hunting strategy within the tree canopy [39]. Its large, laterally positioned eyes may enable it to detect prey movement in the visually cluttered environment of the canopy. A detailed examination of the retinal topography of *A. subcristata* would aid in interpreting this finding, as would behavioural studies testing their visual acuity and response to prey movement.

*Circus assimilis* also stood out in its orbital convergence measurements (figure 8) and in the relative size of its optic lobes (figure 9). Harriers differ from the other raptors sampled in our study in their foraging behaviour and (as discussed above) somewhat resemble *Elanus* kites. Whereas the majority of falcons and hawks hunt by perching, soaring or hovering, harriers engage in a fairly unique strategy known as quartering: low, continuous forward flight over tall grasses and marshes with the beak pointed downwards [70]. To detect prey while quartering, harriers appear to rely not just on visual cues, but also on auditory cues. As shown in laboratory and field experiments by Rice [74], northern harriers (*Circus hudsonius*) can localize sounds with similar precision to barn owls. There has also been a recent suggestion that several *Circus* species, like owls, may have asymmetric tympanic regions ([75]; see also, [76]). A single neurophysiological study by Calford *et al*. [77] indicated that the *C. assimilis* does not have the same spatial encoding for elevation as the barn owl, but anatomical descriptions or data are lacking. More convergent orbits could be advantageous for the final phase of prey capture and/or calibrating the auditory space map, in an analogous fashion to that observed in the barn owl [78]. The enlargement of the optic lobes also could reflect other changes. First, the surface area of the optic lobe could be larger due to the expansion of the underlying inferior colliculus, the auditory centre of the midbrain that is expanded in owls [79] and contains the spatial auditory map [77,80]. Second, interactions between the optic tectum and inferior colliculus are important for spatial localization of sounds [80], which could have driven an increase in optic tectum size (or conceivably optic tectum and inferior colliculus). Histological comparisons of optic lobe structures are required to test these possibilities.

An important caveat in the interpretation of avian cranial anatomy and endocasts is highlighted by the extent of sexual size dimorphism between our two collared sparrowhawk (*Accipiter cirrocephalus*) specimens. Sexual size dimorphism is common among all birds of prey, including owls, typically with larger females. The degree varies across species, with the greatest differences found among *Accipiter* and *Falco* species [65,81]. Females of species like the collared sparrowhawk and other *Accipiter* species can be as much as twice the size of males [81]. By contrast, sexual dimorphism in other species we examined, such as the black-shouldered kite (16% difference in body mass, [81]), is much lower. Although the absolute size of our two sparrowhawk specimens is different, the sexes were located in similar positions relative to the allometric line, so the size correction would limit the confounding influence of sexual size dimorphism. Nevertheless, the variation between our two sparrowhawks suggests that the use of a single data point for studies of the avian endocast or cranial anatomy can be problematic, particularly when allometric effects (disproportionate change with size) is not taken into consideration. We therefore urge the use of more than one specimen, and preferably of known sex, of any species used in comparative endocast studies whenever it is possible.

## 5. Conclusion

Behavioural shifts between nocturnal and diurnal activity patterns have occurred frequently throughout avian evolution, but as our data show, these shifts are not always associated with major changes in skull or brain morphology. This has significant implications for inferring the activity patterns of extinct species, as well as managing the sensory requirements of rare, difficult-to-observe birds. While large relative reductions in orbit dimensions and/or optic lobe sizes probably reflect nocturnality [23,82], more subtle changes in neuroanatomy that cannot be inferred from osteology also enable more sensitive vision and nocturnal activity patterns. An equally important conclusion from our study is that hawks exhibit more diversity in sensory system anatomy than what is often assumed. Harriers have long been considered auditory specialists based on behavioural studies (e.g. [74]) and cranial tympanic region anatomy [75,76], but our data suggest that their vision might also differ from that of other hawks. We have only sampled a small subset of accipitrid and falconid species and as highlighted by Potier [83,84], within these families, there is diversity in retinal anatomy, reliance on olfactory cues, diet and foraging methods, such that a corresponding diversity in neuroanatomy is not surprising. To date, relatively little work has been published on sensory abilities, or sensory system anatomy in either family, with the notable exception of [83,84]. Based on our data, accipitrids, in particular, may be a useful family in which to examine further the correlated evolution of foraging behaviour and nervous system anatomy (both central and peripheral) that could prove useful in interpreting the fossil record more accurately.

## Data Availability

The raw computer tomography slices for all species studied, and three-dimensional reconstructions of skulls and endocasts, will be made publicly accessible on MorphoSource (project P918) upon publication of this manuscript. The raw data and phylogeny, as well as the R code to replicate all analyses and scatterplot figures, are publicly available on github (https://github.com/VWeisbecker/LWK_Visual). The data are provided in the electronic supplementary material [85].
